# A comparative analysis of road and vehicle qualities as factors of road traffic carnage in Nigeria

**DOI:** 10.1186/s12889-023-17089-2

**Published:** 2023-11-06

**Authors:** Onyenekenwa Cyprian Eneh, Andy Okosun, Idu R Egbenta, Nicholas I Obi, Martin C Oloto, Obinna Ubani, Chinemelum A Eneh, Cosmas I Eneonwo

**Affiliations:** 1 Institute for Development Studies, Enugu Campus, University of Nigeria, Nsukka, Enugu State Nigeria; 2 Department of Urban and Regional Planning, Enugu Campus, University of Nigeria, Nsukka, Enugu State Nigeria; 3 Department of Estate Management, Enugu Campus, University of Nigeria, Nsukka, Enugu State Nigeria; 4 Department of Architecture, Enugu Campus, University of Nigeria, Nsukka, Enugu State Nigeria; 5 Department of Agricultural Economics, University of Nigeria, Nsukka, Enugu State Nigeria; 6 Centre for Logistics & Transport Studies, University of Port Harcourt, Rivers State, Nigeria

**Keywords:** Environmental health risks, Public health hazards, Road quality, Vehicle quality, Road traffic Crashes, potholes, Financial burdens, casualties

## Abstract

**Background and objective:**

Carnage on roads is a growing concern in Nigeria. Over 27 persons, equivalent to more than 4 families, die daily from road traffic crashes. Two direct factors of a road crash are road quality and vehicle quality. To interrogate and compare both factors to road traffic accidents, the longitudinal study regressed secondary data on death tolls against road quality and vehicle quality.

**Materials and methods:**

Data on the estimated number of vehicles imported into Nigeria (1992–2021) served as the indicator of vehicle quality on Nigerian roads. The longitudinal study regressed secondary data on death tolls (2013–2019) against road quality (2006–2019) and vehicle quality (1992–2021).

**Results:**

Results showed that road quality is degenerating as well as vehicle quality in Nigeria, resulting in increase in the number of road traffic crashes and the attendant death tolls. For every 1% decrease in road quality, death tolls from road traffic crashes in Nigeria increased by 0.00642% at 5% significance, and for every decrease in vehicle quality, death tolls from road traffic crashes in Nigeria increased by 0.327% at 5% significance.

**Conclusion:**

The study recommended increased advocacy on the sanctity of life and the need for all tiers of government to prioritize policy and implementation of improving the road quality and vehicle quality to reduce road traffic crashes and save lives on Nigerian roads.

## Significance

Growing road traffic crashes account for the death of more than 4 Nigerian families daily. This study analyzed road and vehicle qualities as the major factors of the carnage on Nigerian roads. Findings showed that degenerating road and vehicle qualities are majorly responsible for increase in number of road traffic crashes and attendant death tolls. The study is significant in highlighting the need for increased advocacy on the sanctity of life and the need for tiers of government to prioritize policy and its implementation to improve the road and vehicle qualities to reduce road traffic crashes and save lives and valuable resources in Nigerian.

## Introduction

Transport, which serves to convey people and goods from one place to another, is an important element in economic development and a keystone of civilization. Transport provides or improves access to different locations for people and businesses. It facilitates social and economic interactions. Road transport dominates other modes of movement in Nigeria. Rail transportation dwindled and air transport is unaffordable to the poor masses in Nigeria. Waterways are neglected and poorly spread for inland transportation, and leaders are playing politics with the development of seaports [1].

With a population of 200 million people and an average family size of 6 persons, Nigeria has over 33 million families. There are 1.7 million vehicles in Nigeria, with one vehicle serving over 117 people. Therefore, roads are very essential in Nigeria. Yet, most of them are in poor shape and conditions, inaccessible, too narrow, not developed, and full of potholes. Streets lack functional light for plying the roads at night [2, 3].

Brand-new vehicles are unaffordable to individuals because of galloping inflation and the dwindling exchange rate against the Naira. Only the government affords new vehicles; individual Nigerians have resorted to second-hand (*tokumbo*) vehicles. Before their importation into Nigeria, the vehicles had been involved in serious accidents and adjudged as irreparable by insurance companies. They are shipped to developing countries, including Nigeria, where they are refurbished and sold as *tokumbo.* These poor-quality vehicles reduce vehicle quality on Nigerian roads. The more they are in number, the lower the vehicle quality plying Nigerian roads. Poor road quality and poor vehicle quality combine to form the direct factors of road crashes, which have become growing public health hazards and environmental health risks that inflict deaths, disability and financial burdens. Consequently, socio-economic activities and development limp. Poor road quality and poor vehicle quality combine to cause most road crashes which are the leading cause of death in adolescents and people in their prime age in Nigeria [4].

Road crashes and attendant death tolls occur at an alarming rate in Nigeria. In 6 months (October 2017 to March 2018), about 2,600 Nigerians died in road traffic accidents. Twenty thousand (20,000) vehicles are involved in road traffic accidents every day in Nigeria, leading to a loss of more than 27 lives or more than 4 families daily to road traffic accidents alone [5].

The proportion and an absolute number of traffic fatalities witness an upsurge in several developing countries, but there is a downward trend in industrialized nations. The differential is more than 20% [1]. There was about 37 road traffic crashes every day in January-March 2022 in Nigeria. Road traffic carnage rose by 21.9% between 2014 and 2015, 2.6% between 2016 and 2017 and 1.66% between 2018 and 2019 [6]. There is a similar rising trend in road traffic deaths in Nigeria 7% between 1990 and 1992, 6.1% between 1993 and 1996, 5.6% between 1997 and 1999, 2% between 2000 and 2001, 5.3% between 2002 and 2005, 3.15% between 2006 and 2011, 2% between 2012 and 2014, and 1.4% between 2015 and 2017. Although there was a 1.82% decrease in the number of crashes in October-December 2021, the number of lives lost to road crashes January-March period of 2022 increased by 11.02%. A total of 3,345 road crashes were recorded between January and March 2022, giving an average of 37 road crashes per day. There is a 1.33% rise in the number of roads crashes January-March 2022. About 26% of the 3,345 road crashes recorded between January and March of 2022 were classified as fatal cases, 62.8% were serious cases, and only 374 (11.2%) of the cases were categorized as minor. Nigeria lost a total of 1,834 lives to road traffic crashes between January and March 2022. Male adults accounted for 77.8% of this figure, while female adults were 15.2%. More female children were killed than male children. By way of comparison, 1,652 lives were lost to road crashes between October and December 2021, while 1,834 lives were lost between January and March 2022 – indicating an 11.02% increase in lives lost to road traffic accidents in the succeeding quarter. And, the number of lives lost to road crashes in the January-March 2022 period is higher than those of every quarter of 2021. From the more than 11,800 road traffic casualties that occurred in Nigeria during the fourth quarter of 2021, about 10,200 were injured, while 1,700 were registered deaths [7].

The estimated number of used vehicles imported into Nigeria, as an indicator of vehicle quality, decreased by 73% from 110,715 to 1992 to 30,000 in 1994, increased further by 1,173% from 7,858 to 1997 to 100,000 in 2001, and increased further by 7,506% from 69,411 to 2016 to 760,543 in 2021 [8–12] (Table 1). This showed that vehicle quality degenerated as the number of imported used vehicles increased.

**Table 1:** Estimated number of used vehicles imported into Nigeria

Year	Used vehicles
2021	760,543***
2020	
2019	474,300***
2018	
2017	154,194**
2016	89,411**
2015	112,195**
2014	210,195**
2013	238,193**
2012	228,979**
2011	
2010	
2009	
2008	
2007	
2006	
2005	
2004	
2003	
2002	59,286*
2001	100,000*
2000	50,000*
1999	
1998	22,858*
1997	7858*
1996	
1995	
1994	30,000*
1993	57,143*
1992	110,715*

Source: **** *Statistica* [8]; *** NBS [9]; ** Ukonze, Nwachukwu, Mba, Okeke & Jiburum [10]; Saleh [11], Green-Simms [12]; * Fisa, Musukuma, Sampa et al. [13]

Note: The missing figures are not available due to paucity of data in developing countries. Authors were of the opinion that their non-inclusion would not significantly affect the outcome of the regression

Accidents do not just happen, but they are caused. Two direct factors of road crashes are road quality and vehicle quality. 95% (95%) of second-hand vehicles imported into Nigeria are “accidented” vehicles [13]. Other causes are lack of proper driving education and poor driving behaviour, overload, speed, drunken driving, failure to use provided safety devices, inclement weather, poor vehicle maintenance, dangerous and reckless driving or road violation, fatigue, and use of mobile driving devices and gadgets while driving [3, 14, 15]. This situation, which gives cause for worry, prompted the longitudinal study which set out to regress death tolls from road traffic crashes against road quality and vehicle quality to establish and compare the involvement of both factors in road traffic accidents and the resultant carnage on Nigerian roads.

The study also aimed to update information on older studies. It analyzed current data on road and vehicle qualities as the major factors of the carnage on Nigerian roads. It highlighted the need for increased advocacy on the sanctity of life and the need for all tiers government to prioritize policy and its implementation to improve the road and vehicle qualities to reduce road traffic crashes and save lives and valuable resources in Nigeria.

## Materials and methods

### Materials

The data on road quality were sourced from University of Oxford [5] and Nigeria Customs [7]. Data on death tolls and the estimated number of vehicles imported into Nigeria (1992–2021) were sourced from *Statistica* [8]; NBS [9]; Ukonze, Nwachukwu, Mba, Okeke & Jiburum [10]; Saleh [11], Green-Simms [12]; Fisa, Musukuma, Sampa et al. [13] and Azami-Aghdash, Sadeghi-Bazarghani, Heydari, Rezapour & Deralkhshani [14] as an indicator of vehicle quality on Nigerian roads.

Source: University of Oxford [5] and Nigeria Customs [6].

### Regression analysis

The longitudinal study regressed death tolls (2013–2019) against road quality (2006–2019) (Table 2) and vehicle quality (1992–2021) (Table 3). To investigate the relationship between the variables, secondary data on death tolls were regressed against road quality and against vehicle quality at 5% level of significance. According to Taylor [14], regression analysis estimates the relationships between dependent and independent variables. It is useful for modeling, forecasting and extrapolating the future relationships between the variables. It can be a simple linear regression (SLR), multiple linear regression (MLR) or non-linear regression (NLR), with SLR and MLR being the most common. NLR takes care of datasets with non-linear relationship.

**Table 2:** Quality of roads in Nigeria

Year	Score	Rank
2019	2.5	131
2018	2.4	133
2017	2.5	129
2016	2.6	127
2015	2.71	125
2014	2.68	125
2013	2.66	127
2012	2.77	115
2011	2.72	121
2010	2.39	128
2009	2.55	112
2008	2.32	115
2007	2.21	113
2006	2.41	92

**Table 3:** Estimated number of road traffic deaths in Nigeria

Year	Traffic deaths
2019	41,693
2018	41,008
2017	40,325
2016	39,802
2015	38,749
2014	38,703
2013	37,831
2012	36,948
2011	36,572
2010	39,757
2009	38,996
2008	37,759
2007	37,853
2006	37,662
2005	37,445
2004	37,400
2003	37,774
2002	38,361
2001	37,094
2000	35,748

Source: *Statistica* [8]; NBS [9]; Ukonze, Nwachukwu, Mba, Okeke & Jiburum [10]; Saleh [11], Green-Simms [12]

A correlation analysis measures the degree of association or strength of relationship among variables and reveals the patterns within a dataset. The end result in is in numerical output between − 1 and + 1. Results close to + 1 indicate a positive correlation. Results close to -1 indicate a negative correlation. A positive correlation means that as a variable increases, the other increases also. A negative correlation means that as a variable increases, the other decreases. An output near 0 indicates a less meaningful relationship between the variables [15].

## Results and discussion

From Table 4, the R-squared value of 66.06% (Prob > F = 0.0175) showed that the model was statistically significant.

**Table 4:** Model summary of Quality of vehicles, quality of roads and road traffic deaths

Source	SS	df	MS
Model	5068190.4	2	2534095.2
Residual	2609752.93	3	869917.644
Total	7677943.33	5	1535588.67
F(2, 3) =2.91			
Prob > F =0.1982			
R-squared =0.6601			
Adj R-squared =0.4335			
Root MSE =932.69			

Table 5 showed that for every decrease in road quality, estimated road deaths increased by 0.18642%, and this was significant at 5%. This confirmed the report of Onyemaechi & Ofoma [4] on increasing rates of incidence, morbidity and mortality of road traffic accidents due to the poor state of the roads which have not received the warranted attention in Nigeria. Motor vehicle crashes are the leading cause of death in adolescents and people in their prime age. While the Vehicle Inspection Officers (VIOs) accost and impound vehicles for road-unworthiness, no body or institution is held responsible for roads that are not vehicle-worthy. Political representatives of the people are not held accountable for bad roads that rather destroy vehicles and lives than be vehicle-worthy. As a resource-dependent country, Nigeria has political leaders who do not need to tax their citizens because of a guaranteed source of income from natural resources. On the other hand, accountability is not demanded from the leaders for poor services to citizens by the leaders. If the citizens complain, money from the natural resources enables governments to pay for armed forces to keep the citizens in check in repressive, corrupt and badly-managed manner that mostly involve human right abuse [16]. The finding also falls in line with the submission of Oyeyemi & Agumbiade [5] that successive governments in Nigeria had failed to fix the death traps called roads, bringing so much pain to road users. It also confirmed the report of Azami-Aghdash, Sadeghi-Bazarghani, Heydari, Rezapour & Deralkhshani [17] on the rising trend of road traffic carnage from 4,430 to 2014 to 5,400 in 2015, from 5,049 to 2016 to 5,181 in 2017, and from 5,483 to 2018 to 5,574 in 2019. The finding also tallies with the earlier report of Smith, Cambiano, Colbourn, Collins, Graham, Jewell, Lin, Mangal, Manthalu, Mfutso-Bengo, Mnjowe, Mohan, Ngambi, Philips, Revill, She, Sundet, Tamuri, Twea & Hallet [1] that the proportion and an absolute number of traffic fatalities witness an upsurge in several developing countries, but a downward trend in industrialized nations, with more than a 20% differential.

**Table 5:** Quality of vehicles, quality of roads and road traffic deaths

	(1)
VARIABLES	Estimated road deaths
Quality of road	-1.86e-03**
	(5.77e-04)
Used vehicles number	-0.00327
	(0.0117)
Constant	70,807*
	(26,333)
Observations	6
R-squared	0.660

Standard errors in parentheses

*** p < 0.01, ** p < 0.05, * p < 0.1

Table 5 also showed that, for every 1% decrease in the quality of vehicles, estimated road deaths increased by 0.327% at 5% significance. This confirmed the reports of *Dataphyte* [18], *Autojosh* [19], Isa & Sivan [20], Ayetor, Mbonigaba, Sackey & Andoh [21] and Gopalakrishnan [22] on increasing rates of incidence, morbidity and mortality of road traffic accidents due to low-quality vehicles especially *tokunbo* or second-hand vehicles which constitute over 95% of “accidented” vehicles (previously involved in terrible accidents), leading to being so badly damaged that insurance companies adjudged them irreparable. Every year, hundreds of thousands of them are shipped into Nigeria from the United States of America, Italy, Canada, Belgium and Germany. In a country with soaring inflation and dwindling Naira value, only the government can afford brand-new vehicles, while individuals go for *tokumbo* vehicles and internet-enabled gadgets [17, 23–29].

By way of comparison, for every decrease in road quality, estimated road deaths increased by 0.18642%, whereas for every 1% decrease in the quality of vehicles, estimated road deaths increased by 0.327%. Therefore, deteriorating vehicle quality results in higher road traffic death tolls than a corresponding decrease in road quality.

Death tolls from road traffic crashes have serious consequences in terms of depleting present and future manpower and occasioning profound social challenges. Breadwinners have been lost to road traffic crashes, throwing the family into poverty, jeopardizing the chances of good child upbringing and of obtaining sound education and/or stressing the social family network or African extended family system. Heavy financial costs are usually incurred from road crashes by way of repairing the damaged vehicles, treatment of injuries, and burial of deceased victims, as well as over-tasking already distressed health facilities [1].

## Conclusion

The quality of roads dwindles in Nigeria. Besides, vehicle quality degenerates since second-hand vehicles (or *tokumbo* mostly “accidented”, that is, previously involved in a terrible accident and adjudged irreparable by insurance companies) replace the choice of new vehicles which have become unaffordable amid towering inflation and lowering poor exchange rate for Naira. A combination of these factors is the direct cause of unacceptable levels of increase in the number of road traffic crashes and the attendant very high death tolls. Deteriorating vehicle quality results in higher road traffic death tolls than the corresponding decrease in road quality.

Accidents can be prevented by tackling these chief factors, which have not received the warranted attention by the federal, state and local governments. There is a need for increased advocacy on the sanctity of life. There is, also, the need to draw the attention of governments towards addressing the policy enactment and implementation for the improvement of road quality and vehicle quality to reduce road traffic crashes and save lives on roads.

## Data Availability

Materials and data embedded in this work are available from the corresponding author on request at a reasonable time.

## Code Availability

Materials and data are in Microsoft Word with custom code.
